# Isotope‐Enriched Cubic Boron Arsenide with Ultrahigh Thermal Conductivity

**DOI:** 10.1002/advs.202502544

**Published:** 2025-04-09

**Authors:** Jaehoon Kim, Dongwook Lee, Huan Wu, Joon Sang Kang

**Affiliations:** ^1^ Department of Mechanical Engineering Korea Advanced Institute of Science and Technology (KAIST) Daejeon 34120 South Korea; ^2^ Department of Mechanical Engineering Arizona State University Tempe AZ 85287 USA

**Keywords:** ab‐initio calculation, cubic boron arsenide, isotope, thermal conductivity, thermal management

## Abstract

High thermal conductivity materials are critical for advanced thermal management applications. The semiconductor cubic boron arsenide (c‐BAs) has drawn significant attention due to its ultrahigh thermal conductivity. In this study, high‐quality isotope‐enriched cubic boron arsenide (c‐^10^BAs and c‐^11^BAs) crystals are synthesized to further enhance the thermal conductivity of c‐BAs and measured a room temperature thermal conductivity of 1500 W m^−1^ K^−1^ for the c‐^11^BAs. This value is the highest thermal conductivity for isotope‐enriched c‐BAs reported so far. The experimental study, together with ab initio calculation, verifies the high quality with reproducibility of the crystals. The exceptionally high thermal conductivity of the isotope‐enriched BAs, combined with their semiconductor properties, holds significant potential for improving thermal management in semiconductor devices and electronics packaging applications.

## Graphical abstract

The high‐quality isotope‐enriched cubic boron arsenide crystals that enhance the thermal conductivity of c‐BAs are synthesized and measured at a room temperature thermal conductivity of 1500 W m^−1^ K^−1^ for the c‐^11^BAs. This value is the highest thermal conductivity among known semiconductor materials. The experimental study, together with ab initio calculation, verifies the high quality with reproducibility of our crystals.

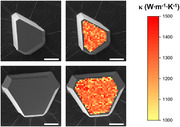

## 1 Introduction

Thermal management is an essential technology for a wide range of electronic devices, including power electronics and optoelectronics.^[^ ^1^, ^2^, ^3^ ^]^ A common approach to achieving efficient thermal management involves utilizing materials with high thermal conductivity to effectively spread heat from localized hot spots. A material with thermal conductivity higher than that of copper (400 W m^−1^ K^−1^) is considered to be a high thermal conductivity material, but there is limited choice of materials that exceed that value.^[^ ^4^ ^]^ Diamond has the highest thermal conductivity (2200 W m^−1^ K^−1^) among known bulk materials. However, high synthesis cost, chemical inertness, and slow growth rate hinder the practical application of diamond.^[^ ^5^ ^]^ On the other hand, graphite suffers from highly anisotropic thermal conductivity because of the weak van der Waals bonding between each layer, resulting in low cross‐plane thermal conductivity.^[^ ^6^ ^]^ Therefore, for efficient thermal management, an alternative high‐thermal conductivity material is necessary.

Cubic boron arsenide (c‐BAs) has attracted significant interest due to its ultrahigh thermal conductivity. First‐principles calculations estimate the thermal conductivity of BAs to be ≈2000 W m^−1^ K^−1^, assuming only three‐phonon scattering—the lowest order phonon‐phonon interaction.^[^ ^7^ ^]^ However, when four‐phonon scattering is included, the calculated thermal conductivity decreases to a range of 1300–1400 W m^−1^ K^−1^.^[^ ^8^, ^9^ ^]^ These theoretical predictions have been experimentally confirmed, establishing BAs as one of the highest thermal conductivity materials among known semiconductors.^[^ ^5^, ^10^, ^11^ ^]^

In addition, c‐BAs exhibits high ambipolar carrier mobility, distinguishing it from other high‐thermal conductivity materials.^[^ ^12^ ^]^ Theoretically, c‐BAs possesses exceptionally high hole mobility (2100 cm^2^ V^−1^ s^−1^), surpassing that of silicon (450 cm^2^ V^−1^ s^−1^) and common III‐V semiconductor materials such as GaN (40 cm^2^ V^−1^ s^−1^) and GaAs (400 cm^2^ V^−1^ s^−1^). High ambipolar mobility of 1600 cm^2^ V^−1^ s^−1^ was experimentally confirmed, highlighting its potential for p‐type semiconductor applications.^[^ ^13^, ^14^ ^]^ Furthermore, this material demonstrates lower thermal boundary resistance with typical semiconductors and metals due to its phonon band structure.^[^ ^15^ ^]^ For instance, a GaN‐on‐BAs structure exhibits a thermal boundary conductance (TBC) of 250 MW m⁻^2^ K⁻¹, significantly higher than that of a GaN‐on‐diamond structure. The combination of high thermal conductivity, mobility, and superior TBC makes c‐BAs a promising candidate for efficient heat dissipation in advanced electronic devices.

Previous studies have focused on enriching boron isotopes to further enhance the thermal conductivity of c‐BAs.^[^ ^16^, ^17^ ^]^ Despite these efforts, experimental measurements have recorded only minimal differences in thermal conductivity between isotope‐enriched and natural c‐BAs (c‐^nat^BAs). Most experimental results report thermal conductivity values in the range of 1100–1300 W m^−1^ K^−1^, regardless of isotopic composition. Given that isotope‐enriched crystalline solids typically exhibit higher thermal conductivity compared to their natural counterparts,^[^ ^18^, ^19^ ^]^ these previous results suggest that impurities may have a more pronounced influence than isotope composition in the c‐BAs samples. Furthermore, significant variations in thermal conductivity have been observed among c‐BAs crystals synthesized using identical crystal growth methods,^[^ ^20^, ^21^ ^]^ even within the same batch. Local defects within individual crystals have also led to considerable deviations in measured thermal conductivity,^[^ ^12^ ^]^ complicating precise comparisons between natural and isotope‐enriched c‐BAs.

We recently reported a highly reproducible c‐BAs synthesis using a transitional metal as a catalyst.^[^ ^22^ ^]^ Here, in this study, we expand our synthesis technique to isotope‐enriched c‐BAs (c‐^10^BAs and c‐^11^BAs) to further increase the thermal conductivity of c‐BAs. The impurity concentrations of the synthesized c‐^10^BAs and c‐^11^BAs samples were characterized using Raman spectroscopy and compared to previous studies, and the free‐charge carrier concentration in our samples remained at exceptionally low levels. By using time‐domain thermoreflectance (TDTR) measurements, we systematically measured the thermal conductivity of c‐^10^BAs and c‐^11^BAs as well as c‐^nat^BAs. The maximum thermal conductivity of the isotope‐enriched BAs reached ∼1500 W m^−1^ K^−1^, surpassing the previously reported experimental values of 1300 W m^−1^ K^−1^. Additionally, ab initio calculations were employed to theoretically study the thermal conductivity of isotope‐enriched c‐BAs with c‐^nat^BAs. A comparison between the experimental data and theoretical calculation revealed that the experimentally measured values exceeded those calculated by ab initio models. These findings highlight the potential of isotope‐enrichment strategies, combined with optimized synthesis techniques, to achieve superior thermal properties in c‐BAs, thereby advancing its applicability for high‐performance thermal management.

## 2 Results and Discussion

**Figure 1:**
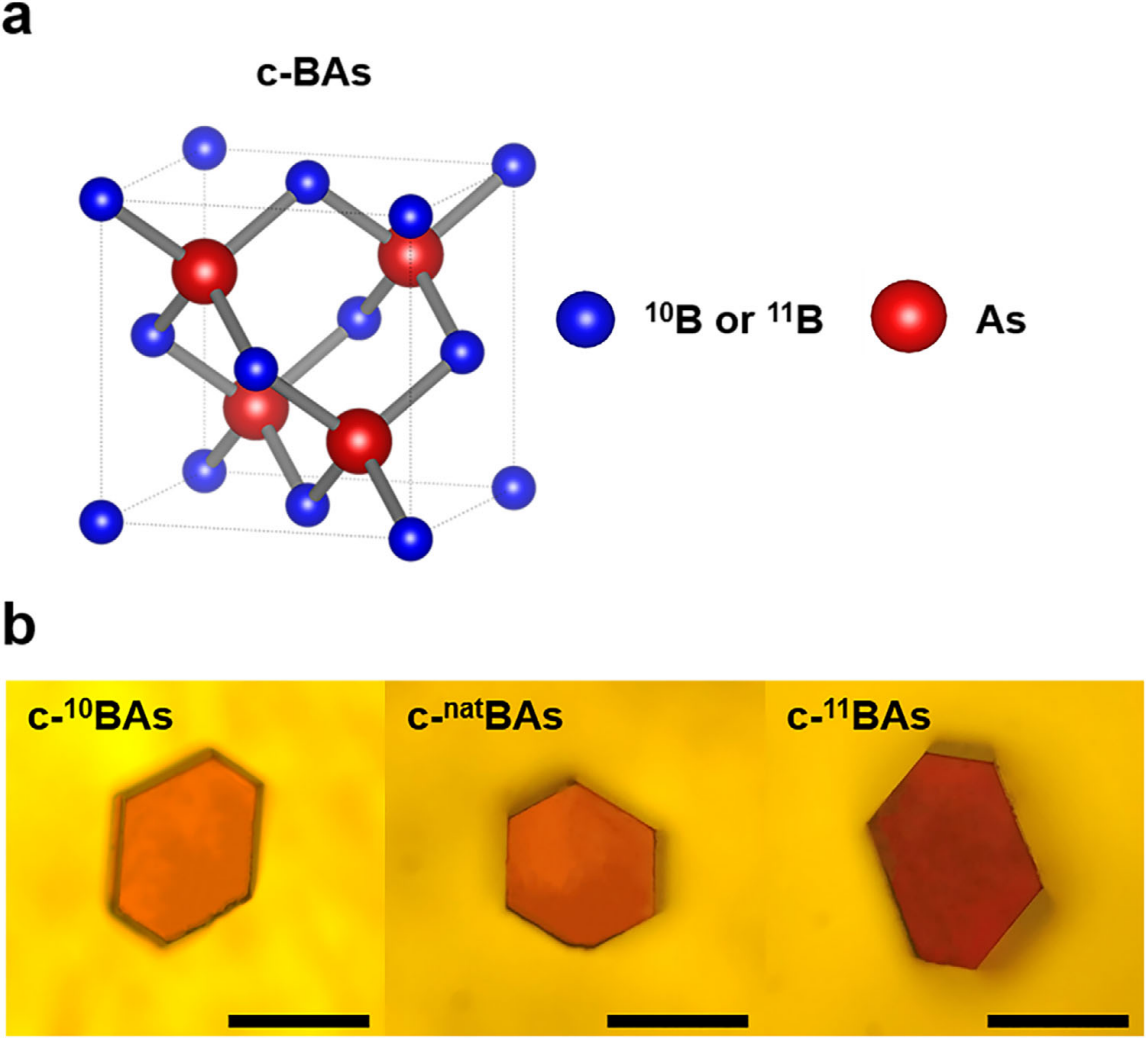
Schematic image of the lattice structure and morphology of cubic BAs with respect to the three different boron isotopes. a) Unit cell structure of cubic BAs. b) Optical images of cubic BAs crystal. Scale bar: 50 µm.

We synthesized high‐quality c‐^nat^BAs, c‐^10^BAs, and c‐^11^BAs using a modified chemical vapor deposition method. Cubic BAs has a zinc blende crystal structure in an $F\bar{4}3M$ space group with a primitive cell containing two atoms. The lattice structure of synthesized c‐BAs with respect to different boron isotopes is shown in **Figure** **1**a. The structure features tetrahedral bonding, where each boron atom is covalently bonded to four arsenic atoms, and each arsenic atom to four boron atoms. Since arsenic exists as a single isotope element, the isotope effect that affects thermal conductivity in c‐BAs is solely determined by boron. Naturally occurring boron (^nat^B) consists of two stable isotopes, 19% of ^10^B, and 81% of ^11^B. This results in an average atomic mass of 10.81 for the ^nat^B. Figure 1b shows optical images of three different c‐BAs isotope crystals. The majority of the crystals exhibit a semi‐transparent reddish hue, with dimensions ranging from a few hundred micrometers (100 to 400 µm) and a consistent morphology, predominantly hexagonal or semi‐hexagonal.

**Figure 2:**
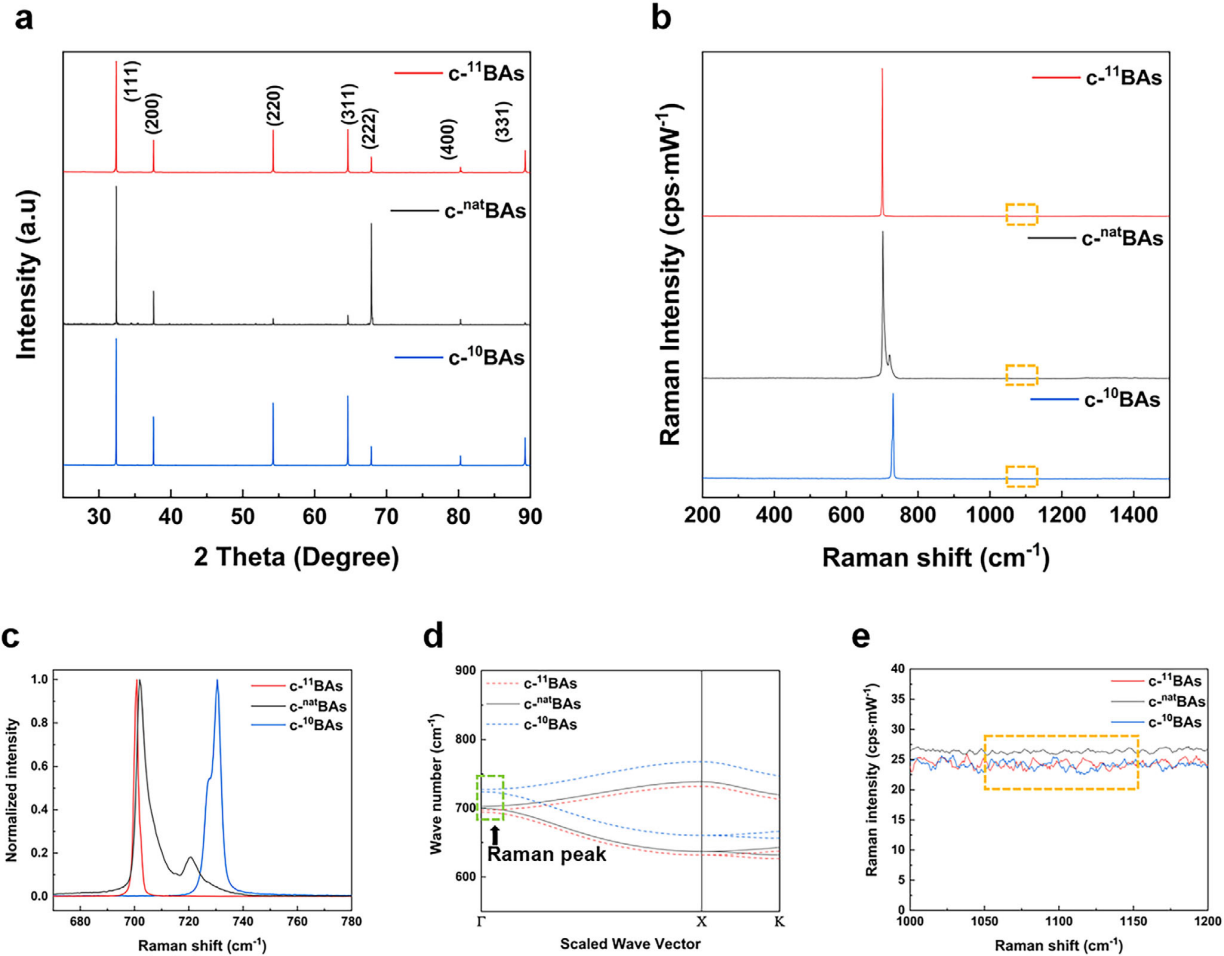
Cubic BAs crystal characterization with respect to the three different boron isotopes. a) Powder X‐ray diffraction (P‐XRD) patterns of the c‐BAs. All three types of isotope c‐BAs show the preferred orientation of (111). b) Measured Raman spectra of the c‐BAs with three types of boron isotope. c) Normalized Raman peak intensity of three types of isotope c‐BAs. d) Calculated phonon dispersions for c‐^10^BAs, c‐^11^BAs, and c‐^nat^BAs at 300K. The green dashed line shows the optical phonon frequency of the c‐BAs at the 1st Brillouin zone center. e) Range of frequency between 1050 to 1150 cm^−1^ where the integration of Raman intensity relates to electronic Raman scattering, illustrated by the yellow dashed line in b). The intensity in counts per second is calibrated by the power of the 633 nm laser excitation in mW units.

To verify the presence of other crystalline phases besides c‐BAs, powder X‐ray diffraction (P‐XRD) analyses employing a Cu Kα radiation source were conducted. As shown in **Figure** **2**a, no peaks corresponding to other crystalline substances were observed, and identical peaks were confirmed for c‐^10^BAs, c‐^11^BAs, and c‐^nat^BAs in each batch. The presence of sharp peaks and the narrow full‐width at half maximum (FWHM) indicate the high crystallinity of these materials.

Also, the lattice constant extracted from the XRD measurement was 4.78 Å, which remained consistent for all three c‐BAs crystals with different boron isotopes. In particular, peaks along the (111) crystal orientation were strongly observed for all three types of c‐BAs, suggesting that the c‐BAs crystals are mainly oriented in that direction. This result is consistent with previous theoretical calculations, which indicate that c‐BAs preferentially grows along the (111) orientation because (111) has the highest surface density, meaning the (111) surface is the most closely arranged, ultimately resulting in the lowest surface energy of the plane.^[^ ^23^ ^]^ Besides, as shown in Figure 1b, the optical images of all three types of c‐BAs clearly reveal a hexagonal shape, and this further confirms that the exposed surface is the (111) plane.^[^ ^24^ ^]^

Next, Raman spectra of the different boron isotope c‐BAs were obtained. The Raman peak position is influenced by the optical phonon frequency at the center of the Brillouin zone (Γ point), which is sensitive to isotope composition. Figure 2b shows the Raman spectra of c‐^nat^BAs, c‐^11^BAs, and c‐^10^BAs from 200 to 1500 cm^−1^. In this range, no other Raman peaks are observed except for those of the c‐BAs, but slightly different Raman peak spectra near 700 cm^−1^ are exhibited by each c‐BAs. In Raman spectroscopy, heavier atoms tend to exhibit Raman peaks at lower shifts because the lattice vibrational frequency of a mode is inversely related to the square root of the reduced mass of the system.^[^ ^25^ ^]^ As the atomic mass increases, the reduced mass also increases, resulting in a lower vibrational frequency and, consequently, a lower Raman shift.

In Figure 2c, single Raman peaks are observed for c‐^10^BAs and c‐^11^BAs, whereas two Raman peaks are observed for c‐^nat^BAs. In typical crystals, translational symmetry limits Raman‐active modes. However, in c‐BAs, isotope disorder and the significant vibrational energy splitting between boron isotopes break this symmetry. This disruption leads to Brillouin zone folding, revealing new vibrational modes. As the isotope concentration shifts, the Raman spectra transition from a single peak in isotope‐enriched crystals to dual peaks in isotope‐mixed crystals. For instance, c‐^nat^BAs display two Raman peaks at ≈700.8 and 718.9 cm^−1^ due to the coupling and mass differences between ^10^B and ^11^B isotopes. Increasing ^10^B concentration shifts these peaks to higher frequencies as lighter atoms oscillate at higher frequencies.^[^ ^26^ ^]^ In Figure 2c, from experiments, the Raman shifts of each isotope c‐BAs crystals are presented, with distinct peaks for c‐^nat^BAs at 702 and 719 cm^−1^ originating from the isotopes of boron, c‐^10^BAs at 729 cm^−1^ and c‐^11^BAs at 700 cm^−1^. It should be noted that the c‐^10^BAs exhibited a peak position of 729 cm^−1^, slightly deviating from the calculated value of 730 cm^−1^, and displayed a shoulder that was not observed in the c‐^11^BAs. This small discrepancy between theoretical prediction and experiment was attributed to the slightly lower isotope enrichment of the ^10^B powder compared to the ^11^B powder.

To validate our experimental result with theory, we calculated the phonon dispersion of three sets of c‐BAs based on first‐principle calculations. As shown in Figure 2d, the Raman peak positions matched well with the optical phonon frequencies at the Γ point for each of the isotope‐enriched c‐BAs, and the values closely corresponded to the calculated ones. Additionally, the finite FWHM in the Raman spectra of the c‐BAs indicates a pronounced higher‐order anharmonic phonon scattering,^[^ ^27^ ^]^ as the lowest‐order anharmonic scatterings are suppressed by the large acoustic‐optical bandgap and do not contribute to the Raman linewidth.

Phonon‐impurity scatterings could be one potential factor to reduce thermal conductivity. To assess the impurity levels in our sample, we examined the Raman spectral range associated with electronic Raman scattering. In the literature, Raman peaks with a Fano line shape often appear in the range of 1050–1150 cm^−1^, which arises from the interaction between an optical phonon and an electronic excitation of free charge carriers introduced by impurities.^[^ ^10^, ^21^ ^]^ The extent of this interaction depends on the charge carrier concentration and can be evaluated through the integrated Raman intensity within this range. Figure 2e presents a zoomed‐in view of the Raman spectra from 1050  to 1150 cm^−1^, showing no Raman peaks within this spectral range. With the same Raman intensity unit, our measurements validate that all samples, including both isotope‐enriched c‐BAs and c‐^nat^BAs, consistently demonstrate a very small integrated Raman intensity of less than 3%. This indicates that all of the c‐^10^BAs, c‐^11^BAs, and c‐^nat^BAs have similarly low charge carrier concentrations compared to previous studies. Based on these results, we assume our samples contained low impurity concentrations, making phonon‐isotope scattering more dominant than phonon‐impurities scattering.

**Figure 3:**
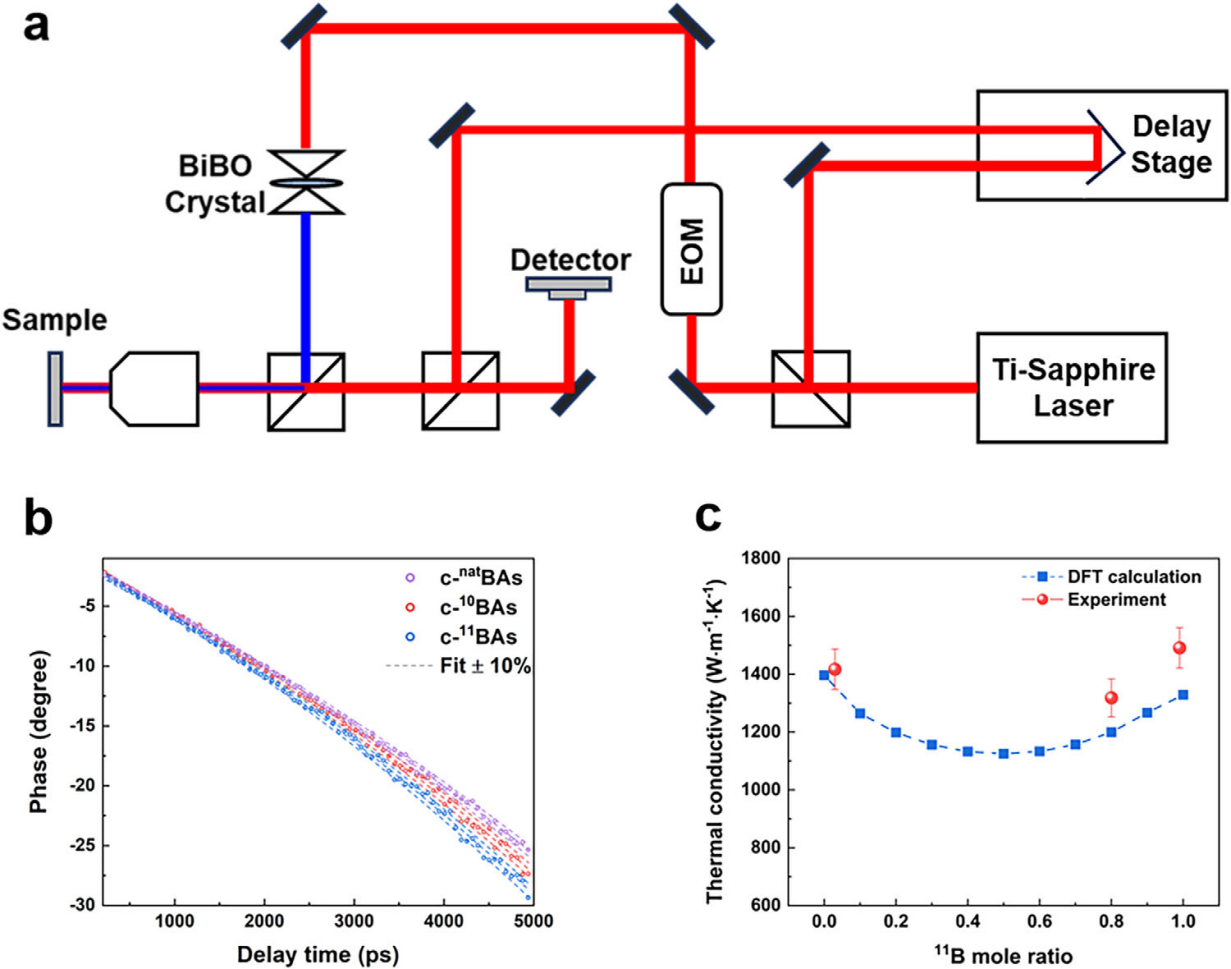
Thermal conductivity measurement of isotope‐controlled cubic BAs crystals. a) Schematic of the TDTR method. b) Typical TDTR phase data and fitting of BAs crystal. The solid line is the best fit of the experimental data, and dashed lines are the calculated curves using thermal conductivity changed ±10% from the best fit. c) Measured and calculated thermal conductivity with respect to different ^11^B mole ratios.

Next, the thermal conductivity of the c‐^10^BAs, c‐^11^BAs, and c‐^nat^BAs was characterized using the TDTR method. TDTR can measure the thermal properties of samples with high reliability and spatial resolution^[^ ^28^, ^29^, ^30^ ^]^ (Supporting Information). A schematic of the TDTR is illustrated in **Figure** **3**a. Phase data were used to extract the thermal conductivity of the c‐BAs crystals. In Figure 3b, the phase data and best‐fit curves are shown along with dashed lines created via varying thermal conductivity by ± 10%, indicating the phase data has enough sensitivity for thermal conductivity extraction. The measured thermal conductivity for c‐^10^BAs was 1420 W m^−1^ K^−1^, and for c‐^11^BAs, it was 1500 W m^−1^ K^−1^, which shows improvements of 8% and 13%, respectively, compared to c‐^nat^BAs (1320 W m^−1^ K^−1^). It should be noted that our crystal exhibited the highest thermal conductivity among isotope‐enriched c‐BAs reported to date.

To theoretically explain the increase in thermal conductivity based on the isotope composition of the c‐BAs, we calculated the lattice thermal conductivity using ab initio approaches based on density functional theory.^[^ ^31^ ^]^ Both three‐phonon and four‐phonon scatterings were considered. The isotope scatterings are determined by quantum perturbation theory.^[^ ^32^ ^]^ Figure 3c shows the thermal conductivity of the c‐BAs as a function of isotope content, providing a comparison between experimental measurements and theoretical calculations. According to the ab initio calculation results, the thermal conductivity of the isotope‐enriched c‐BAs was 10–16% higher than that of c‐^nat^BAs. While these trends align with the experimentally measured thermal conductivities as a function of isotope concentration, the experimental absolute values were ≈5–10% higher than the theoretical calculations, potentially due to variability arising from the choice of pseudopotentials in the density functional theory calculations. Compared with diamond and c‐BN, the isotope effect on thermal conductivity in the c‐BAs is relatively weak. Isotope‐enriched samples of diamond and c‐BN exhibit thermal conductivity enhancements of 45% and 80%, but the c‐BAs showed only a marginal increase in thermal conductivity at room temperature.

**Figure 4:**
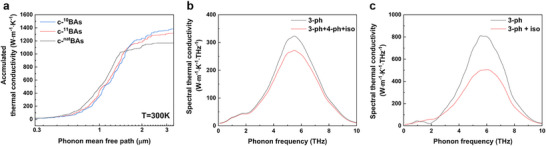
Ab initio calculation of thermal conductivity of c‐BAs. a) Calculated accumulated thermal conductivity as a function of the phonon mean free path for c‐^10^BAs, c‐^nat^BAs, and c‐^11^BAs at 300K. b) Spectral thermal conductivity of c‐BAs without considering four‐phonon scattering. c) Spectral thermal conductivity of c‐BAs with considering four‐phonon scattering.

To investigate the weak isotope dependence of thermal conductivity in c‐BAs, we calculated the spectral‐dependent phonon properties shown in **Figure** **4**. Figure 4a shows the accumulated thermal conductivity as a function of phonon mean free path spectra at room temperature for c‐^10^BAs, c‐^11^BAs, and c‐^nat^BAs. In the c‐BAs, the majority of heat was carried by phonons with mean free paths ranging between 0.5 and 2 µm, regardless of isotope enrichment. The phonon mean free path showed very limited variations with changes in isotope content, indicating that phonon‐isotope scattering is weak and has little impact on the phonon mean free path. To understand why phonon‐isotope scatterings have small impact on the thermal conductivity of c‐BAs, we compare the spectral thermal conductivity obtained from theoretical calculations with and without four‐phonon scattering, as shown in Figure 4b,c, respectively. Isotope scattering follows the Rayleigh scattering mechanism, where scattering rates depend on phonon frequency and density of states. When four‐phonon scattering is included, the reduction in thermal conductivity from isotope‐enriched to natural isotope abundance is small. In contrast, without four‐phonon scattering, the reduction is significant. This indicates that without four‐phonon scattering, intrinsic anharmonic phonon scattering in c‐BAs is weak, making phonon‐isotope scattering more impactful. When four‐phonon scattering is included, intrinsic anharmonic phonon scattering becomes stronger, reducing the relative impact of phonon‐isotope scattering on thermal conductivity. When only three‐phonon scattering is considered, the thermal conductivity of isotopically pure c‐BAs increases by 40%.^[^ ^33^ ^]^ However, when four‐phonon scattering is additionally accounted for, the increase in thermal conductivity is only 16%. The weak isotope dependence of thermal conductivity in c‐BAs is attributed to intrinsic anharmonic scattering being stronger than isotope scattering when both lowest‐order and higher‐order anharmonicity are considered.

**Figure 5:**
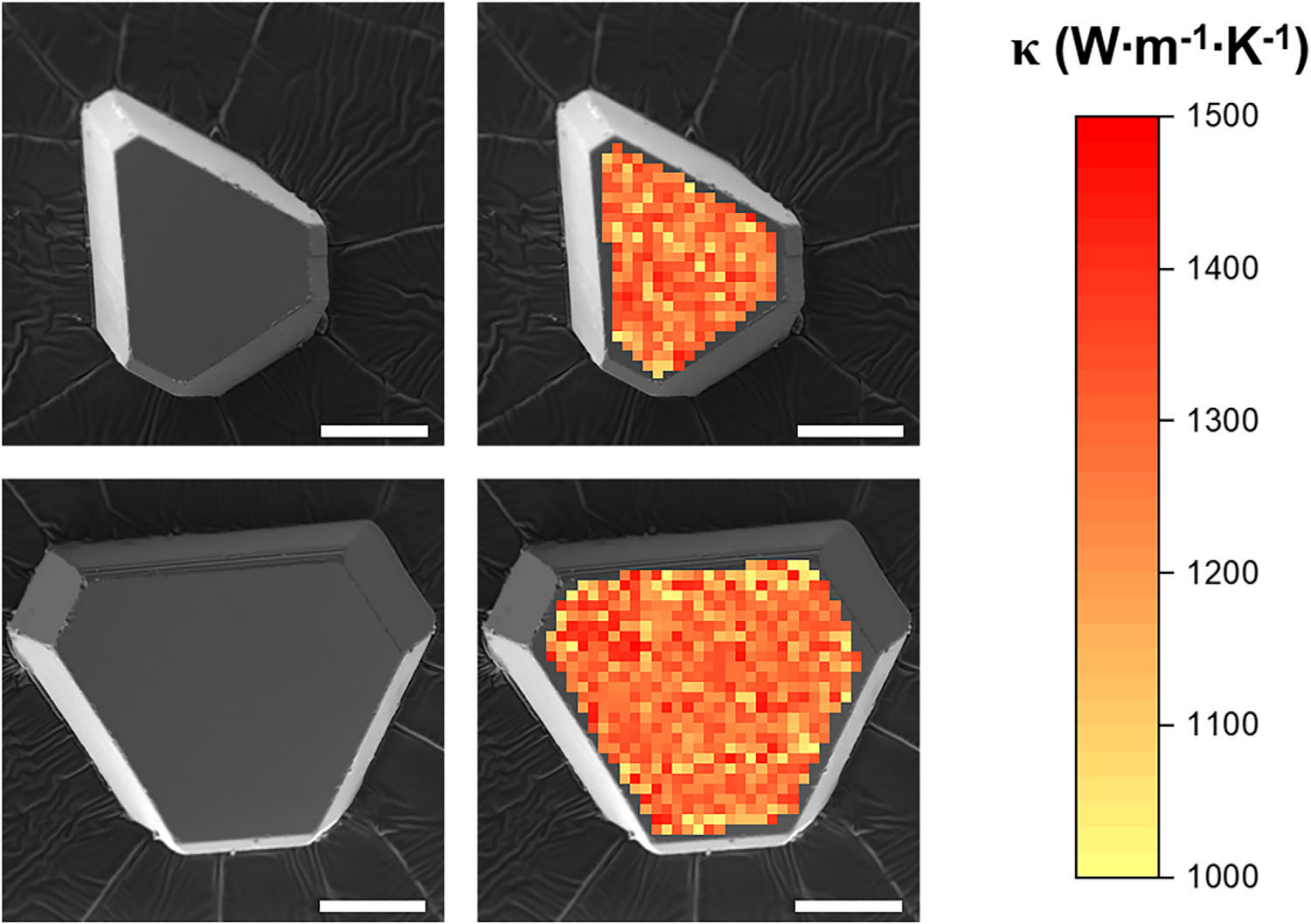
Thermal conductivity mapping of isotope‐enriched c‐BAs crystals. a) Scanning electron image of a selected c‐BAs crystal for thermal conductivity mapping. b) 2D thermal conductivity mapping through entire crystal. Scale bar: 40 µm.

Next, thermal conductivity mapping of the synthesized BAs crystals was carried out to investigate the uniformity of thermal conductivity throughout the entire crystal. Prior to mapping the thermal conductivity, a TDTR sensitivity analysis was performed to select a delay time to minimize sensitivity to the thermal interfacial conductance. A fixed delay time of 300 ps was chosen for the thermal conductivity mapping. The detected phase signal was used to calculate thermal conductivity at each point on the sample. The thermal conductivities measured across both isotope‐enriched c‐BAs crystals exceeded 1000 W m^−1^ K^−1^, as shown in **Figure** **5**. The results show that both isotope‐enriched crystals had uniformly high thermal conductivity across the entire crystal surface.

**Figure 6:**
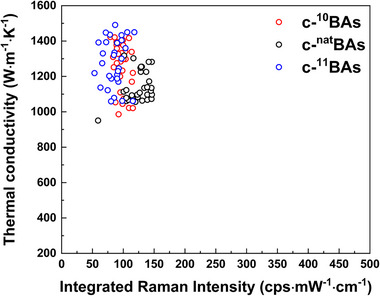
Reproducibility of high thermal conductivity c‐BAs crystal. Relationship between Integrated Raman intensity and measured thermal conductivity for thirty randomly selected c‐^10^BAs, c‐^11^BAs, and c‐^nat^BAs crystals.

To evaluate the reproducibility of the synthesized c‐^11^BAs, c‐^10^BAs, and c‐^nat^BAs crystals in this study, 30 crystals were randomly selected from each batch of c‐BAs, and their thermal conductivity and integrated Raman intensity were systematically measured. This was achieved by comparing the Raman intensity measured in the 1050–1150 cm^−1^ range with the thermal conductivity values obtained through TDTR measurements. As shown in **Figure** **6**, it was observed that among the 30 randomly selected crystal samples more than 95% of all the c‐^nat^BAs, c‐^10^BAs, and c‐^11^BAs had a thermal conductivity of over 1000 W m^−1^ K^−1^, and showed there was enhanced peak thermal conductivity in the c‐^10^BAs and c‐^11^BAs compared with the c‐^nat^BAs. Also, all the integrated Raman intensity values for the c‐^10^BAs, c‐^11^BAs, and c‐^nat^BAs were within 50–150 cps·mW^−1^·cm^−1^, indicating highly reproducible crystal synthesis, and a similarly low phonon‐free carrier scattering rate in all three types of c‐BAs. Based on these results, an increase in the thermal conductivity of c‐BAs crystals due to isotopes was observed when phonon‐impurity scattering was maintained at similarly low levels.

It should be noted that the theoretical calculation predicts that the thermal conductivity of the c‐^10^BAs should be higher than that of c‐^11^BAs. However, experimental measurements showed that the thermal conductivity of the c‐^10^BAs was lower than that of the c‐^11^BAs. This discrepancy is likely attributed to the lower isotope enrichment of the ^11^B source (99%) compared to the ^10^B source (97%).

Nonetheless, enhancement in thermal conductivity due to isotopes was observed in the c‐BAs crystals when phonon‐impurity scattering was maintained at similarly low levels. Furthermore, despite the finite values of integrated Raman intensity in the c‐BAs samples used in this study, the measured thermal conductivity across multiple crystals was higher than the theoretically calculated values. Therefore, it is anticipated that the thermal conductivity of perfectly impurity‐free, isotope‐enriched c‐BAs could be even higher than the values measured in this study.

## 3 Conclusion

In summary, we report the highest thermal conductivity of isotopically enriched c‐BAs crystals and systematically observe the isotope effects of boron on the thermal conductivity of c‐BAs crystals. The measured maximum thermal conductivities in the c‐^10^BAs and c‐^11^BAs were 1420 and 1500 W m^−1^ K^−1^, respectively, which was higher than that of the c‐^nat^BAs at 1320 W m^−1^ K^−1^. Based on the comparison of our experiment with ab initio calculations, we conclude the enhancement in thermal conductivity in isotope‐enriched c‐BAs is relatively weaker than other high thermal conductivity materials, but the maximum value of measured thermal conductivity was higher than the theoretically predicted value. Additionally, highly reproducible crystal synthesis was also confirmed by measuring the thermal conductivity and Raman spectra of a large number of c‐BAs crystals. Our results show that it is possible to achieve c‐BAs thermal conductivity of 1500 W m^−1^ K^−1^ by using the isotope‐enrichment strategy in conjunction with a high‐quality crystal synthesis technique. Isotope‐enriched c‐BAs could be a highly promising candidate for advanced thermal management material in next‐generation high‐performance electronics.

## 4 Experimental Section

### Material Synthesis

Single crystal c‐^10^BAs, c‐^11^BAs, and c‐^nat^BAs were synthesized using a modified chemical vapor transport technique, using platinum as a reaction medium, which acts as a catalyst. The source material of pure crystalline ^nat^B powder (SS Nano) or isotope‐enriched ^10^B powder (Cambridge Isotope), and ^11^B powder (Cambridge Isotope) were mixed with arsenic chunks (Alfa Aesar) at the stoichiometric ratio of B:As = 1:2.1. For the isotope‐enriched boron powders, ^10^B and ^11^B had enrichments of 97% and 99%, respectively. Platinum, which was used as a catalytic layer, was deposited onto an Al_2_O_3_ substrate. Considering the toxic properties of arsenic vapor during reaction, all the source materials were placed in a well‐designed quartz tube. After the materials were placed, the quartz tube was fused under high vacuum (<10^−3^ Pa). The assembly was placed in a 30 cm‐length horizontal tube furnace with the source materials at the hot end, and slowly heated at a rate of 100 K h^−1^ to 1123 K for 2 weeks, then slowly cooled down to 893 K at a rate of 0.5 K h^−1^. After the temperature reached 893 K, the furnace was rapidly cooled down to room temperature. After the reaction, each of the obtained c‐^10^BAs, c‐^11^BAs, and c‐^nat^BAs crystals had a semi‐transparent reddish hue with typical linear dimensions of 300 µm. To get rid of residue, c‐^10^BAs, c‐^11^BAs, and c‐^nat^BAs, the crystal samples were washed out thoroughly with hydrochloric acid, followed by acetone and isopropanol wash.

### Powder X‐Ray Diffraction

Powder X‐ray diffraction measurements were performed with a Rigaku Smartlab using a Cu Kα radiation source. Samples of the synthesized c‐^10^BAs, c‐^11^BAs, and c‐^nat^BAs crystals were separated from the growth substrate and transferred to the zero‐diffraction substrate. The diffractometer was operated at 40 kV and 40 mA. The diffraction patterns were collected from 20 to 90° at a scan rate of 5° min^−1^. After measurement, the diffraction peaks were matched with ICDD material data and identified as c‐BAs. No peaks corresponding to other crystalline substances were observed. In particular, the peak positions for the c‐^10^BAs, c‐^11^BAs, and c‐^nat^BAs were identical, confirming that isotope boron does not affect the lattice structure of the material. As a result, the extracted average lattice constant of the c‐^10^BAs, c‐^11^BAs, and c‐^nat^BAs was ≈4.78 $\dot{A}$.

### Raman Spectroscopy

The Raman spectroscopy measurements were performed with a dispersive Raman spectrometer (ARAMIS, Horiba) under a 633 nm excitation laser. The spectral resolution of the Raman spectra was ≈0.8 cm^−1^ for 633 nm excitation when using 1200 grooves mm^−1^ grating. Also, the spatial resolution of the laser spot was 1.4 µm with a numerical aperture of 0.55/50x microscope objective lens. The Raman spectra used for background intensity were obtained by integrating the Raman shift from 1050 to 1150 cm^−1^. The power of the excitation laser was 3 mW, as measured with a laser power meter (PM100D, Thorlabs). Before measuring the c‐^10^BAs, c‐^11^BAs, and c‐^nat^BAs, the instrument was calibrated using a silicon wafer with a Raman peak at 521 cm^−1^. After the measurement, the intensity in counts per second was calibrated by the power of the 633 nm laser excitation (3 mW) and the exposure time (3 s).

### Thermal Conductivity Measurement

The time‐domain thermoreflectance (TDTR) method was employed for the thermal conductivity measurement of the c‐BAs samples. A Ti‐sapphire laser emits femtosecond laser pulses with an 800 nm wavelength. The emitted laser beam was divided into pump and probe beams using a polarized beam splitter. To modulate the pump beam, it was directed through an electro‐optic modulator (EOM), which was electronically connected to a function generator. The pump beam was sinusoidally modulated with a frequency of 9.8 MHz for our measurements. Then the fundamental frequency of the pump beam was doubled using a bismuth triborate (BIBO) crystal, facilitating second‐harmonic generation to create a 400 nm wavelength pump beam. The probe beam's optical path was adjusted using a mechanical delay stage and a set of retroreflective mirrors, varying the time delay between the arrival of the pump beam and the probe beam on the sample's surface. After passing through the optical systems, the pump and probe beams were aligned coaxially and focused on the sample's surface. The reflected probe beam carries information regarding the thermoreflectance signal, and it was measured with a photodetector and a lock‐in amplifier. The intensity and phase data of the probe beam were analyzed and fit a theoretical heat model to extract thermal conductivity.

### Ab Initio Calculation

The phonon dispersion and lattice thermal conductivity were derived using ab initio methods, with interatomic force constants (IFCs) determined by density functional theory (DFT). The phonon dispersion was determined by diagonalizing the dynamical matrix. The lattice thermal conductivity was determined by solving the phonon Boltzmann transport equations with the full scattering matrix derived from quantum perturbation theory. The three‐phonon, four‐phonon, and isotope scatterings were considered in the scattering matrix. The DFT calculations were performed using the Quantum ESPRESSO package, with projector‐augmented wave pseudopotentials under local density approximation. The irreducible displacement‐force set was determined from DFT with a kinetic energy cutoff of 120 Ry for electronic wavefunctions and a Monkhorst‐Pack grid of 2 × 2 × 2 for a 216‐atom supercell. The IFCs were extracted by fitting the irreducible displacement‐force set using ALAMODE package. Third‐ and fourth‐order IFCs included interactions up to the fifth and second nearest neighboring atoms, respectively. The scattering matrix was computed using an 18 × 18 × 18 mesh for phonon wavevectors in the Brillouin zone.

## Conflict of Interest

The authors declare no conflict of interest.

## Supporting information



Supporting Information

## Data Availability

The data that support the findings of this study are available from the corresponding author upon reasonable request.
