# Supplementary material for: Isotope‐Enriched Cubic Boron Arsenide with Ultrahigh Thermal Conductivity
Source: Adv Sci (Weinh). 2025 Apr 9;12(25):2502544. doi: 10.1002/advs.202502544 (PMC12225002; doi:10.1002/advs.202502544)
Supplement: Supplementary file 1 — Supporting Information [file ADVS-12-2502544-s001.pdf]

ADVANCED SCIENCE
Open Access

Supporting Information

for *Adv. Sci.*, DOI 10.1002/advs.202502544

Isotope-Enriched Cubic Boron Arsenide with Ultrahigh Thermal Conductivity

Jaehoon Kim, Dongwook Lee, Huan Wu and Joon Sang Kang**

Supplementary Materials for

Isotope-Enriched Cubic Boron Arsenide with Ultrahigh Thermal Conductivity

Jaehoon Kim[1+], Dongwook Lee[1+], Huan Wu[2*] and Joon Sang Kang[1*]

Department of Mechanical Engineering,

Korea Advanced Institute of Science and Technology (KAIST), Daejeon, South Korea, 34120

Department of Mechanical Engineering,

[2]Arizona State University, Tempe, AZ, United States, 85287

[+] These authors are contributed equally: J.K and D.L.

*Corresponding author. Email: huanwu@asu.edu, jskang1@kaist.ac.kr

Contents

S1. TDTR sensitivity analysis

S2. Morphology and size distribution of the isotope-enriched boron powder

S3. Energy-dispersive X-ray (EDX) mapping

S4. Structural analysis of c-^{10}BAs, c-^{nat}BAs, and c-^{11}BAs

S1. TDTR Thermal conductivity mapping and sensitivity analysis

Thermal conductivity mapping was conducted using TDTR. The ratio of the in-phase and out-of-phase signal acquired by the lock-in-amplifier is:[1]

$$\frac{V_{in}(t)}{iV_{out}(t)} = \frac{\sum_{q=-\infty}^{\infty} g(q) exp(i2\pi tq/\tau)}{\sum_{q=-\infty}^{\infty} h(q) exp(i2\pi tq/\tau)}$$

For sufficiently small delay time t, the ratio R = V_{in}/V_{out} is proportional to the surface temperature. Thermal conductivity mapping was conducted by fixing the delay time and measuring the signal ratio R across different sample surfaces by moving the sample with micrometer-attached sample stage. Following a sensitivity analysis of TDTR with respect to the delay time, a fixed delay time of 200ps was selected for mapping.

Phase sensitivity to thermal conductivity can be expressed as:[2,3]

$$S_{\phi,k} = \frac{d\phi}{d\ ln\ k}$$

Where ϕ represents the phase and k denotes thermal conductivity. Sensitivity analysis was conducted for the TDTR phase data of c-BAs samples with our experimental conditions.

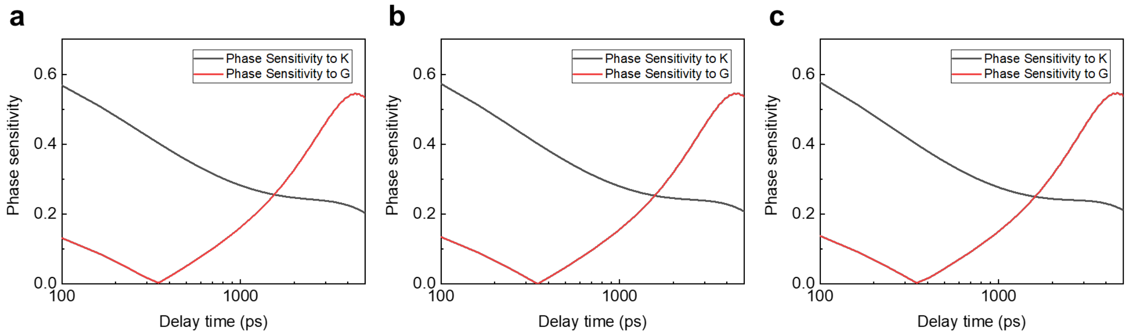

Figure S1. TDTR sensitivity analysis. TDTR phase sensitivity to thermal conductivity K and thermal interfacial conductance G with our experimental conditions for sample a) c-^{10}BAs b) c-^{nat}BAs, c) c-^{11}BAs samples.

S2. Morphology and size distribution of the isotope-enriched boron powder

The particle size distribution of ^{10}B and ^{11}B without grinding are about 9.39 µm and 122 µm, respectively. To minimize the impact of the particle size difference between ^{10}B and ^{11}B during synthesis, we thoroughly ground the powders using a mortar and pestle. As a result, as shown in Figure R4 (c) and (d), the size distribution of ^{10}B and ^{11}B were confirmed to be nearly identical, with average 250 particles of 2.31 um and 2.98 um, respectively.

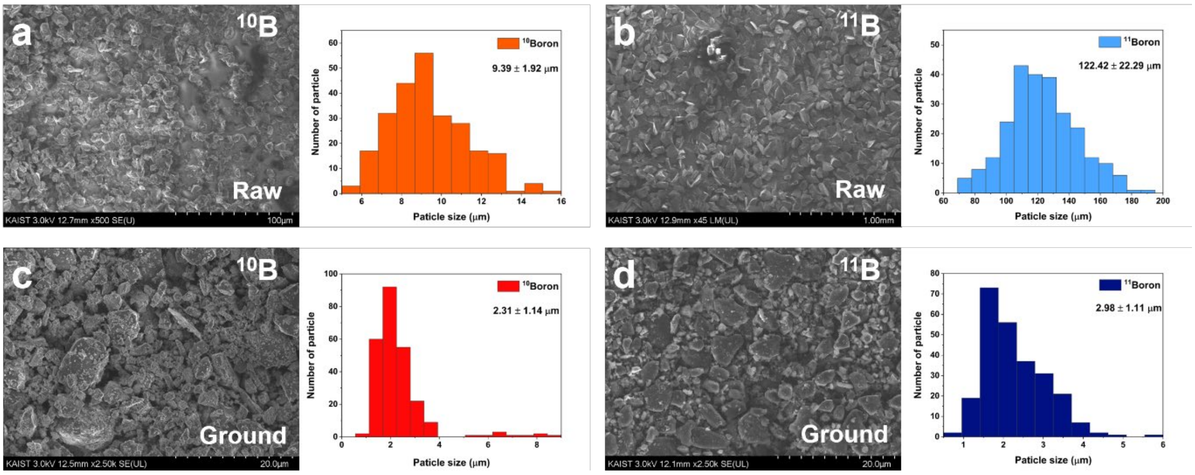

Figure S2. Morphology and size distribution of isotope-enriched boron powders, each for 250 particles (^{10}B and ^{11}B) with and without grinding using a mortar and pestle. a) ^{10}B powder before grinding b) ^{11}B powder before grinding c) ^{10}B powder after grinding d) ^{11}B powder after grinding

S3. Energy-dispersive X-ray (EDX) mapping

EDS X-ray (EDX) mapping was done on randomly picked c-^{10}BAs, c-^{nat}BAs, and c-^{11}BAs sample. As shown in the white dashed line in Figure S3, all the c-BAs samples clearly have evenly distributed Arsenic (As) map with similar signals. Also, we mapped C and Si in c-BAs samples because they could dominantly serve as shallow acceptors, ultimately leading to the p-type conducting behavior. Figure S3 shows that impurities such as C and Si were barely present in the sample, with the C and Si signal acting mainly as background noise[4].

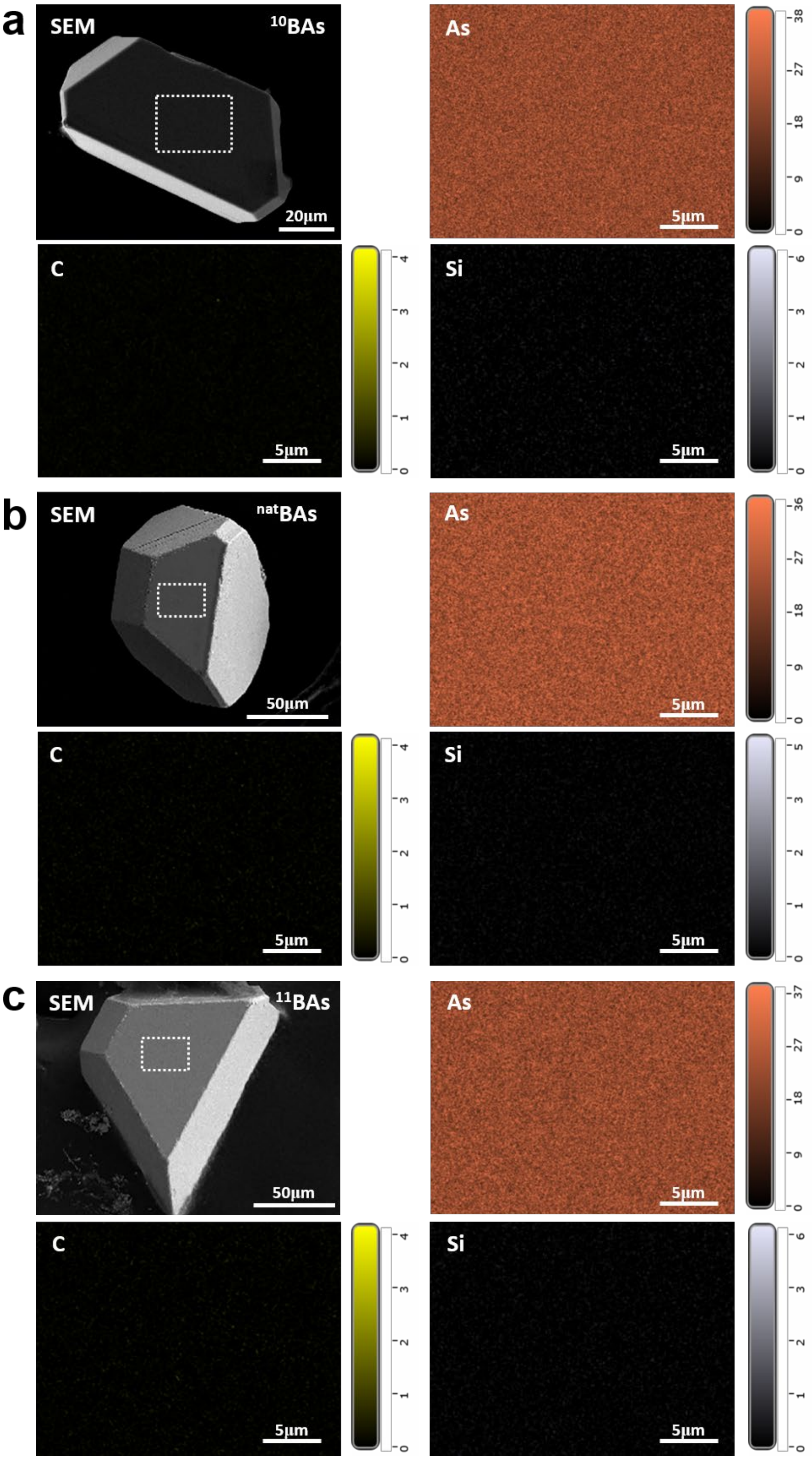

Figure S3. EDS X-ray mapping images. Orange, yellow, and grey dots represent the elements Arsenic (As), Carbon (C), and Silicon (Si), respectively. SEM and impurity mapping for a) c-^{10}BAs. b) c-^{nat}BAs. c) c-^{11}BAs. The dashed line on the surface indicates the target area for element mapping (30 μm x 20 μm). Note that the C and Si map display only background noise.

S4. Microscopic measurements (SEM and TEM) of c-^{10}BAs, c-^{nat}BAs, and c-^{11}BAs

As shown in Figure S4, all the samples exhibited smooth surfaces and hexagonal shapes in their SEM image. Furthermore, over 90% of the c-^{10}BAs, c-^{nat}BAs, and c-^{11}BAs samples showed either hexagonal or triangular shape based on their 30 SEM images. Also, With the same [111] zone axes, we took HRTEM images of c-^{10}BAs, c-^{nat}BAs, and c-^{11}BAs and they all revealed atomically resolved lattices. There were seemingly no exist of structural defect, such as edge dislocations or screw dislocations.

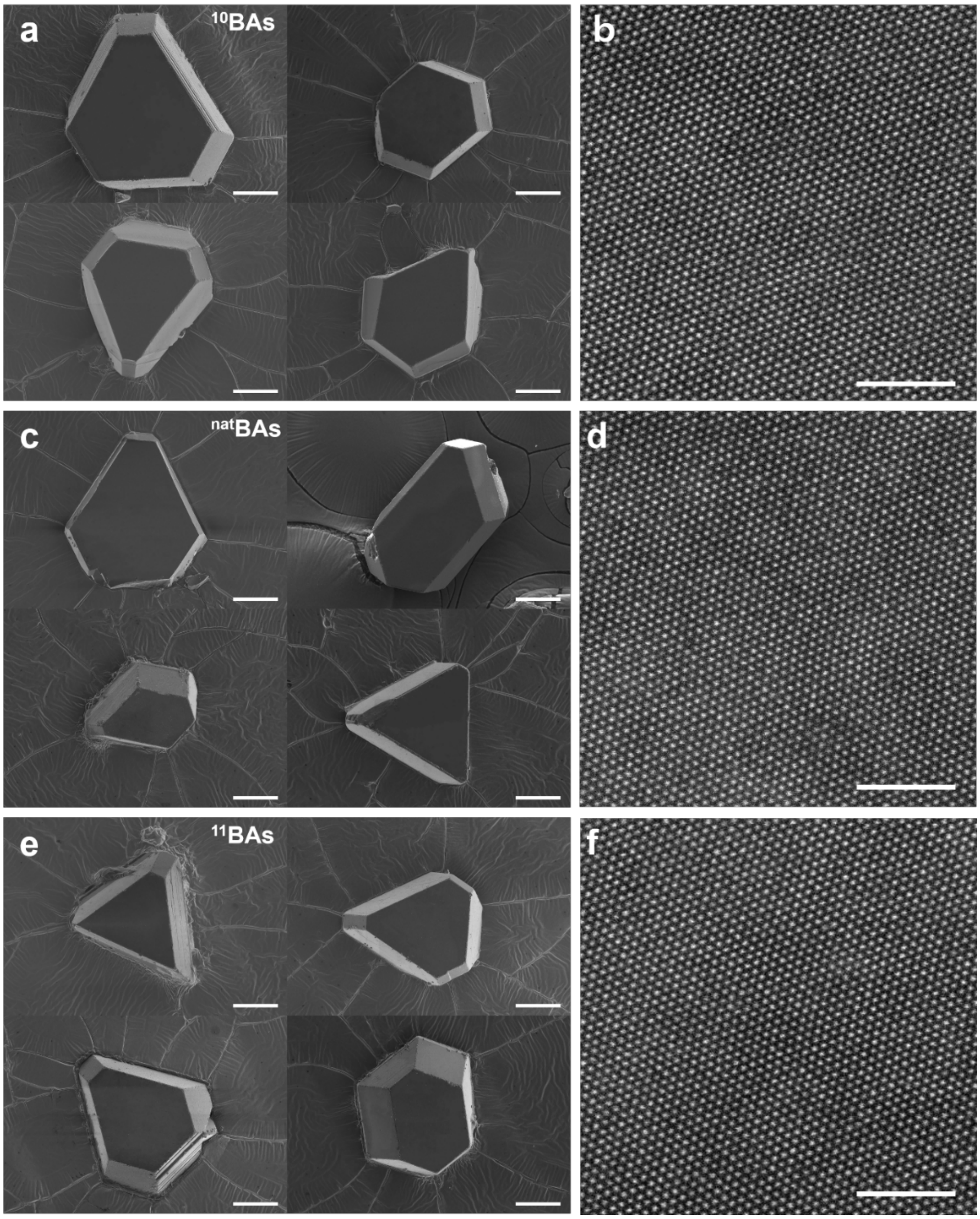

Figure S4. Structural analysis of c-^{10}BAs, c-^{nat}BAs, and c-^{11}BAs. a) SEM images of c-^{10}BAs. b) TEM image of c-^{10}BAs. c) SEM images of c-^{nat}BAs. d) TEM image of c-^{nat}BAs. e) SEM images of c-^{11}BAs. f) TEM image of c-^{11}BAs. (Scale bar for SEM : 50 μm, TEM : 2 nm)

References

[1] S. Huxtable, D. G. Cahill, V. Fauconnier, J. O. White, J. C. Zhao, *Nat Mater* **2004**, *3*, 298.

[2] B. C. Gundrum, D. G. Cahill, R. S. Averback, *Phys Rev B* **2005**, *72*.

[3] A. J. Schmidt, X. Chen, G. Chen, *Review of Scientific Instruments* **2008**, *79*.

[4] X. Chen, C. Li, Y. Xu, A. Dolocan, G. Seward, A. Van Roekeghem, F. Tian, J. Xing, S. Guo, N. Ni, Z. Ren, J. Zhou, N. Mingo, D. Broido, L. Shi, *Chemistry of Materials* **2021**, *33*, 6974.
